# Weight Gain and Fatigue Effect on Nursing Students Performing High-Fidelity CPR Simulation

**DOI:** 10.3390/jcm14217483

**Published:** 2025-10-22

**Authors:** Santiago Morejón Bandrés, José Luis Martin Conty, Begoña Polonio-López, Samantha Diaz-Gonzalez, Cristina Rivera-Picón, Sergio Rodríguez-Cañamero, Juan José Bernal-Jiménez, Joseba Rabanales-Sotos, Miguel Ángel Castro-Villamor, Rosa Conty-Serrano, Ancor Sanz-García, Francisco Martín-Rodríguez

**Affiliations:** 1 Servicio de Urgencias Médicas de Madrid (SUMMA 112), 28045 Madrid, Spain; santimorejon@gmail.com; 2 Faculty of Health Sciences, University of de Castilla-La Mancha, 45600 Talavera de la Reina, Spain; joseluis.martinconty@uclm.es (J.L.M.C.); begona.polonio@uclm.es (B.P.-L.); samanta.diaz@uclm.es (S.D.-G.); cristina.rivera@uclm.es (C.R.-P.); juanjose.bernal@uclm.es (J.J.B.-J.); ancor.sanz@uclm.es (A.S.-G.); 3 Technological Innovation Applied to Health Research Group (ITAS Group), Faculty of Health Sciences, University of de Castilla-La Mancha, 45600 Talavera de la Reina, Spain; 4 Evaluación de Cuidados de Salud (ECUSAL), Instituto de Investigación Sanitaria de Castilla-La Mancha (IDISCAM), 45004 Toledo, Spain; 5 Grupo de Investigación Multidisciplinar en Cuidados (IMCU), Universidad de Castilla-La Mancha, 45004 Toledo, Spain; 6 Faculty of Nursing, University of Castilla la Mancha, 02071 Albacete, Spain; joseba.rabanales@uclm.es; 7 Group of Preventive Activities in the University Health Sciences Setting, University of Castilla-La Mancha, 02071 Albacete, Spain; 8 Valladolid Rural II Health Center (SACYL), 47610 Valladolid, Spain; miguelangel.castro.villamor@uva.es; 9 Faculty of Medicine, University of Valladolid, 47005 Valladolid, Spain; francisco.martin.rodriguez@uva.es; 10 Faculty of Nursing, University of de Castilla-La Mancha, 45005 Toledo, Spain; rosamaria.conty@uclm.es; 11 Advanced Life Support, Emergency Medical Services (SACYL), 47003 Valladolid, Spain

**Keywords:** simulation-based medical education, CPR, nursing, weight, fatigue

## Abstract

**Background/Objectives:** This study aimed to determine the effects of weight gain and fatigue on nursing students performing high-fidelity cardiopulmonary resuscitation (CPR) simulation. **Methods:** A simulation-based research study (with or without a vest) was performed at the Faculty of Health Sciences, University of Castilla la Mancha (Spain), with volunteer nursing students. Vital signs, pain scale, pupillary sizes, analytical parameters, and lifestyle habits were evaluated before, during, and after CPR. The differences between groups were assessed by ANOVA for time and group factors. **Results:** A total of 31 participants met the inclusion criteria, 15 subjects without a vest and 16 subjects with a vest. The ANOVA results revealed that all the variables presented statistically significant time effects, except for glucose. For the group effect, heart rate, lactate, and cortisol presented statistically significant differences between subjects with and without vests. **Conclusions:** Vital signs and physiological variables increased during CPR with vests. This could negatively impact the CPR procedure. The implementation of physical training programs aimed at improving the performance of future health professionals during CPR should be studied.

## 1. Introduction

Cardiac arrest occurs when breathing and/or circulation in a person abruptly and unexpectedly ceases as a result of various underlying causes [1]. From a clinical point of view, it is characterized by the absence of cardiac activity, manifested through a lack of response, the absence of a pulse and the presence of apnea [1]. The interventions aimed at recovering vital functionality in these cases are called cardiopulmonary resuscitation (CPR). CPR is a procedure carried out by health professionals in emergency and emergency services, mainly, in addition to other health services, in patients during cardiac arrest. Some of the essential elements that favor high-quality CPR include an adequate depth of compression and total thoracic expansion, in addition to a certain compression frequency range [2]. For this reason, it has been proven that quality CPR increases patient survival; however, CPR quality declines over time due to rescuer fatigue from performing chest compressions in most health care professionals [3,4,5,6].

The aging of staff is one of the determining elements when executing critical procedures, such as CPR [7,8]. Skill and experience, when prolonged and effective chest compressions are performed, are vital to ensure adequate cerebrovascular perfusion in patients and can be influenced by variables such as physical fitness, weight, body mass index (BMI) or fatigue [2,9,10,11]. This issue is particularly relevant in the global context of an aging healthcare personnel [12,13] and the widespread use of shift work in hospital settings [14,15], both of which are associated with reduced physical capacity and increased fatigue. These factors can degrade CPR performance and sustainability over time.

The physical condition of rescuers is a determining factor that directly influences the quality of CPR. Muscle strength and endurance play crucial roles in maintaining effective chest compressions for prolonged periods of time, whereas physical fatigue, which can appear in the early phases of CPR, limits the ability to sustain a proper technique [2,5,12]. In addition, this type of fatigue manifests itself more quickly in rescuers with less physical capacity, significantly affecting the quality of compressions and increasing the variability in their execution [2,3,4,5]. This decrease in performance not only has an immediate effect on the effectiveness of the intervention but could also compromise patient safety by altering the uniformity of the procedure implemented [6].

Similarly, another essential variable that could lead to an incorrect performance of CPR is an increase in BMI [5,12]. However, a high BMI or greater weight of the rescuers favors exercising a greater depth in the chest compressions due to body mass, optimizing the effectiveness of these methods [2,5,16]. However, a high BMI can accelerate the onset of fatigue, compromising the quality of maneuvers [10]. Therefore, we must highlight the importance of combining an adequate technique with good physical shape and an optimal BMI to guarantee the correct execution of CPR. This phenomenon is also correlated with sex, with fatigue appearing earlier in women than in men because of lower weight, BMI and physical fitness [12]. This problem can be intensified in high-demand contexts, in which physical and emotional exhaustion are transformed into additional elements that put the quality of interventions at risk [12]. Therefore, the main objective of this study was to assess how additional weight (achieved by the wearing of a weighted vest) affects rescuer fatigue in nursing students during simulated CPR.

## 2. Materials and Methods

### 2.1. Study Design

A simulation-based research study (with or without a vest) was carried out, including students over 18 years of age with basic knowledge of basic life support, following the criteria of the American Heart Association (AHA) and/or the European Resuscitation Council (ERC). The participants were selected from the degree programs in nursing, occupational therapy, and speech therapy of the Faculty of Health Sciences of the University of Castilla-La Mancha (Talavera de la Reina, Spain). All participants provided informed consent, and the study was approved by the Ethics Committee of the Castilla-La Mancha Health Service (SESCAM), under the number 178013/113, in compliance with the Declaration of Helsinki.

The study was developed in Talavera de la Reina from September 2023 to June 2024. It included 31 nursing students from the University of Castilla-La Mancha, assigned to groups randomly by XLSTAT^®^ BioMED 14.4.0 software (Microsoft Inc., Redmond, WA, USA). A final sample of 31 participants was selected, which were distributed into two groups for the simulation of CPR (15 participants from the control group without a vest and 16 participants with a vest). Before performing the CPR simulations, all participants had attended a hands-on seminar with manikins. The selected participants were between 18 and 34 years old and had prior knowledge of basic resuscitation maneuvers.

### 2.2. Population

The sample size was calculated assuming an alpha risk of 0.05 and a beta risk of 80%. To detect a minimum difference of 15% between the two groups and a standard deviation of 30% obtained from previous studies, it was estimated that 7 subjects per group would be necessary, for a total of 14 subjects.

**Figure 1:**
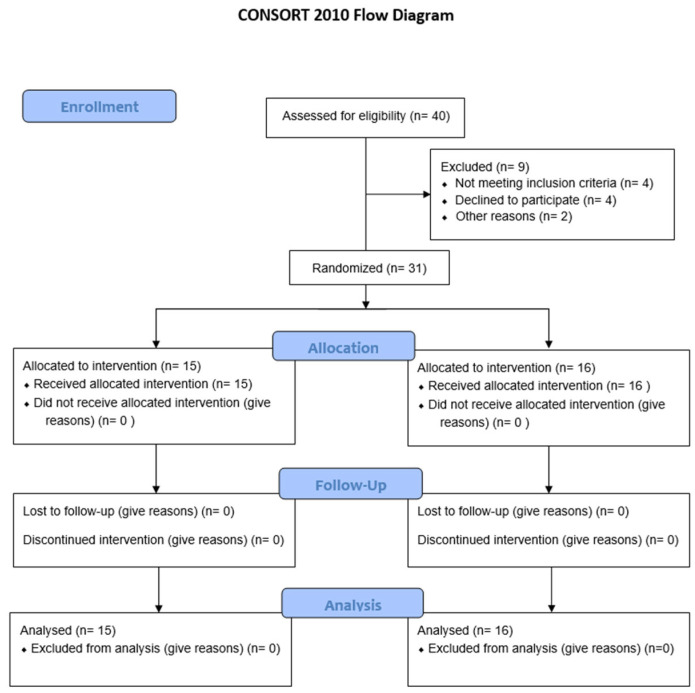
Consort 2010 Flow Diagram.

The following exclusion criteria were proposed: did not have training accredited by the AHA or ERC in CPR; a resting heart rate (HR) > 120 bpm or <35 bpm, a systolic blood pressure > 160 mmHg or <80 mmHg, diastolic > 95 mmHg; blood glucose < 65 mg/dL; severe visual/hearing impairments or functional disorders; oxygen saturation < 92%; electrocardiogram with abnormalities (arrhythmia or ST changes); a body temperature > 38 °C; diagnosis of epilepsy, infection, or ongoing treatment; and a BMI > 40 kg/m^2^ or acute skin or immune system diseases. The CONSORT 2010 flow diagram summarizes participant eligibility, allocation, follow-up, and analysis, and is presented below (Figure 1).

### 2.3. Study Protocol

Each participant underwent a medical examination. This examination included a focused anamnesis, vital signs (weight, height, body mass index (BMI), oxygen saturation, blood pressure, heart rate, temperature), pain scale (Visual Analog Scale (VAS)), pupillary size (NPi-300 Pupillometer (NeurOptics, Inc., Irvine, CA, USA)), and basic analytical determinations (cortisol, glucose and lactic acid). These variables were monitored before starting the test and at the end of the study. In addition, the International Physical Activity Questionnaire (IPAQ) was used to evaluate the level of activity of the subjects, along with a questionnaire on alcohol, tobacco, coffee, and energy drink consumption. Room temperature, humidity and lumens were collected before the start of the test. VAS was performed as previously described [17]; briefly, pain intensity was assessed via a numerical rating scale, where participants rate their whole body (independently of location) pain from 0 to 10, with 0 representing no pain and 10 representing the worst pain imaginable. Pupillary size has recently been correlated with anxiety during clinical simulation [18].

For the test, we designed a scenario in which all the volunteers had to perform CPR for 20 min without stopping, following the quality standards of the AHA (30 chest compressions followed by two ventilations), differentiating both groups by wearing or not wearing a vest with weight [9,12]. The vest group was equipped with a 10 kg vest and two wrist straps of 1.5 kg each (GERonTologic simulator (GERT) suit). The rescuer received continuous feedback regarding the compression depth, compression rate, and hand position.

Before (as part of the initial medical examination), immediately after, immediately after, and at 60 min and 90 min after the CPR simulation, the pain scale, heart rate, respiratory rate, oxygen saturation, heart rate, systolic, diastolic, and mean blood pressure, and body temperature were measured. Pupillary sizes were measured before and after the CPR simulation. Finally, lactate, cortisol, and glucose were measured before, during CPR at minutes 5, 10 and 15, immediately after, and at 60 min and 90 min after the CPR simulation. These final measurements were performed using the Epoc^®^ Blood Analysis System (Siemens Healthcare GmbH, Erlangen, Germany) for glucose and lactate, and cortisol was measured with an Affias-6 (© Boditech Med, Inc., Chuncheon-si, Gang-won-do, Republic of Korea), with a measurement range of 80–800 nmol/L.

### 2.4. Data Analysis

A univariate analysis was conducted between the subjects who used a vest and those who did not use it for all the variables analyzed. In addition, an analysis of the temporal dynamics was carried out for those variables that had more than one measure in time using a two-way repeated measures ANOVA to assess time and group effects (vest vs. no vest). The data analysis was performed with the statistical software SPSS, v. 24 (SPSS Inc., Chicago, IL, USA), with a significance level set at 0.05.

### 2.5. Ethical Considerations

The study subjects were informed about the general objectives of the study, and informed consent was obtained. The study was approved by the Ethics Committee in Clinical Research of Talavera de la Reina (Toledo) with the number 178013/130.

## 3. Results

### 3.1. Descriptive Results

The final sample of 31 participants consisted of 10 men (32.3%) and 21 women (67.7%). If we look at the variable of wearing the weight vest or not, there were a total of 15 subjects who did not wear it: men, *n* = 3 (20%); women, *n* = 12 (80%); and 16 subjects who did: men, *n* = 7 (43.8%); and women, *n* = 9 (56.2%). The mean age of the subjects who did not wear the vest was 26.4 ± 9.3 years, and that of the subjects who did wear the vest was 22.3 ± 3.8 years (Table 1).

In the descriptive results obtained from the sample (Table 1), no significant differences were observed between study participants based on the shift during which the protocol was performed, morning or afternoon shifts (*p* = 0.396). Therefore, the shift in which CPR did not affect participants’ performance, regardless of other variables analyzed.

Regarding their body composition, the 15 students (48.4%) who did not wear the vest had a BMI of 21.7% ± 3.1, and the 16 students (51.6%) who wore the vest had a BMI of 23.5% ± 3.8. Similarly, the students who did not wear the vest weighed 59.3 kg ± 9.8 kg, with a size of 165 cm ± 0.2 cm, and the 16 students who wore the vest weighed 69.2 kg ± 17 kg, with a size of 171 cm ± 0.1 cm (Table 1).

In terms of lifestyle variables, 22 (71%) of the 31 participants reported not consuming alcohol, and the remaining 9 (29%) did. With respect to tobacco consumption, 24 (77.4%) identified as nonsmokers, and 7 (22.6%) smoked. With respect to coffee intake, 9 (29%) did not consume it, and 22 (71%) did consume it. Finally, 24 (77.4%) of the participants did not consume Red Bull^®^. However, 7 (22.6%) did consume it (Table 1).

According to the IPAQ physical activity questionnaire, of the 31 participants, 18 (58.1%) were in the sedentary group, 7 (22.6%) were in the active group, and 6 (19.3%) were in the sports group (Table 1).

Finally, when all the analytical parameters and vital signs were considered, only cortisol presented increased baseline levels in the vest group (*p* = 0.042).

Next, an analysis of the temporal dynamics was conducted for the different variables that had more than one measure in time. For these variables, a two-way ANOVA of repeated measures was performed for time and group with or without a vest.

### 3.2. Analysis on the Basis of Vital Signs

First, an analysis of vital signs was performed for SPO_2_, HR, and blood pressure (Table 2). During the performance of the CPR maneuver, significant differences over time were observed for several of these variables, although no significant differences were detected in the use of the vest, except for HR.

#### 3.2.1. Oxygen Saturation

Significant differences in SpO_2_ over time were recorded between the two groups (F(1, 3) = 4.111; *p* = 0.008). However, no significant differences (*p* > 0.05) were found between the vest and no-vest groups.

#### 3.2.2. Blood Pressure

Regarding the measurement of systolic blood pressure and mean arterial pressure, significant differences were recorded for the time variable (F(1, 3) = 7.789; *p* < 0.001). However, the use of a vest did not result in significant differences (*p* > 0.05). Likewise, if we look at diastolic blood pressure, significant differences were recorded the time variables (F(1, 3) = 3.821; *p* = 0.011), although no differences were obtained in the use of the vest.

#### 3.2.3. Heart Rate

In the analysis of HR, compared to the other vital signs mentioned above, significant differences were obtained (F(1, 3) = 11.01; *p* = 0.001) in the use of the vest by the rescuers during the CPR procedure. The participants who used the vest obtained had higher HR values than those who did not wear the vest. Similarly, HR values were significantly different for the time variable (F(1, 3) = 21.42; *p* < 0.001), indicating a progressive increase in HR during the performance of the CPR protocol, regardless of the group.

#### 3.2.4. Temperature

For body temperature, significant differences were obtained for the time variable (F(1, 3) = 4.485; *p* = 0.005). However, there were no differences between the groups in the use of the vest during CPR.

### 3.3. Analysis of Physiological and Physical Variables

An additional analysis was conducted considering physiological variables specific to the rescuers (Table 3), where significant differences were obtained depending on the use or not of the vest:

#### 3.3.1. Lactate in Blood

For the blood lactate variable, significant differences were recorded for the time variable, such as for the use of the vest (F(1, 3) = 17.7; *p* < 0.001). Similarly, a significant increase in lactate was observed over time (F(1, 3) = 13.51; *p* < 0.001).

#### 3.3.2. Cortisol

The cortisol variable significantly increased in the group that used the vest (F(1, 3) = 6.12; *p* = 0.0141), and a significant increase was reflected in the time variable (F(1, 3) = 11.8; *p* < 0.001).

#### 3.3.3. Glucose

Unlike the other physiological variables, glucose did not significantly differ between the groups (*p* = 0.402), nor did the time variable (*p* = 0.242).

#### 3.3.4. Pupil Size

Regarding the size of the pupil, significant differences were obtained for the time variable in both pupils: the right pupil (F(1, 3) = 16.1; *p* < 0.001) and the left pupil (F(1, 3) = 22.82; *p* < 0.001). However, there were no differences between the groups in the use of the vest during CPR.

#### 3.3.5. Pain Scale (VAS)

Regarding the pain scale, significant differences were obtained for the time variable (F(1, 3) = 3.61; *p* = 0.015). However, there were no differences between the groups in the use of the vest during CPR.

## 4. Discussion

Currently, the aging of health care personnel poses a great challenge, being considered a relevant variable in this context of demanding procedures such as CPR, owing to its high physical, metabolic and stress impact [11,13,19,20]. The effectiveness of the CPR maneuver and its quality can be compromised by the age and physical condition of the rescuer [21,22]. In this context, the aging of health care personnel and the impact of overweight and BMI during CPR are still under debate. Although some studies suggest that an increase in the body mass of rescuers help them perform more effective chest compressions (CCs), due to the depth reached [23,24], others suggest that being overweight increases and intensifies fatigue, negatively affecting the quality of the maneuver [24,25]. Our results highlight that the participants who wore the vest and, therefore, had greater body weight to mobilize when performing CPR did not show improved performance. This could be due to an increase in the effort and fatigue of the rescuers, who were not able to maintain an optimal quality of the resuscitation maneuver over time for prolonged periods [26]. These results accentuate its relevance in the context of the aging of health personnel, where it has already been proven that age itself is not a limiting factor, but rather the decline in physical condition, which reduces their ability to maintain optimal performance during the maneuver [27]. 

Our findings demonstrate the impact of additional body weight upon fatigue during CPR, which should be studied in the future to determine the effect on the quality of CPR [26]. The use of a weighted vest has a significant influence on some of the physiological variables and vital signs studied, such as HR, blood lactate, or cortisol. These findings indicate that there is a greater physical and metabolic demand on rescuers who carry out CPR maneuvers. These results, therefore, corroborate what has already been reported in other studies related to performance/physical variables and the quality of CPR performance [2,6,19,28].

In relation to the study variables of blood pressure (systolic, diastolic and mean blood pressure), significant differences were obtained over time, with no differences between the different groups under study. This cardiovascular response was similar between the two groups, in addition to the fact that the use of the vest did not have a significant effect on the variability and regulation of blood pressure during CPR. However, these results differ from the results obtained in previous investigations, in which significant differences were obtained in blood pressure in relation to the execution of physical exercise by rescuers, which was increased in those with a worse physical condition and age [29,30].

The progressive increase in HR during CPR was one of the main results obtained, since significant differences were obtained both for the time variable and between the control group and the group with a vest. This increase in the cardiovascular demand of rescuers who wore the vest reflects that the effort required to maintain an adequate quality of chest compressions intensifies with increasing load. Previous studies have established a direct correlation between rescuer fatigue, with an increase in HR and a worsening of the quality of compressions [4,20,31]. Likewise, the literature already highlights that physical condition of the rescuers is considered a crucial factor for optimal maintenance in the case of having to perform prolonged CPR. This has been related to a worse physical resistance of the rescuers, which is associated with a decrease in the quality of chest compressions caused by the appearance of premature fatigue [5,11,12].

Another significant variable of the study was the increase in the blood lactate level of the rescuers who wore the vest. It is predominantly anaerobic due to the characteristics of the CPR procedure, suggesting increased muscular demand and fatigue, as well as the accumulation of fatigue as the protocol advances [32]. These results are related to those obtained in previous studies, in which a greater increase in blood lactate was associated with intense and sustained physical efforts, such as CPR, in rescuers with worse physical conditions [9,10,32]. Similarly, previous studies have shown that rescuers with better physical conditions have a lower concentration of lactate in the blood during CPR, indicating that resistance training programs for healthcare workers could serve as an effective strategy for increasing the performance and quality of CPR [16].

Regarding the cortisol variable, understood as our marker of physiological stress, we also obtained significant differences both for the time variable and between the control group and the one that used the vest. Previously, how CPR-induced physiological stress responses have been studied because of the cognitive and physical load that the maneuver itself implies; in addition, this response significantly increases with weight of the rescuer [12,33]. In line with this, marked physiological stress responses (including cortisol-related markers) have been documented during simulated high-acuity resuscitation tasks, showing that increasing task demands amplify stress levels and may degrade performance under pressure [34]. Likewise, other investigations have also shown how elevated cortisol levels can compromise decision-making, as well as the accurate execution of CPR, especially in high-demand scenarios and in rescuers with less experience [35,36,37].

This study is not free of limitations. (i) The quality of the CPR, i.e., compression characteristics, was not collected. Therefore, the effect of weight and fatigue on the performance of the study participants, that is, compression depth, compression rate, compression fraction, hands-off time, etc., was not reported. (ii) There was a cortisol baseline difference between groups with and without vests. This could be associated with the increased stress associated with the vest use; however, no anxiety differences were found, as indicated by pupillary size measurements.

## 5. Conclusions

Increased physical demand on rescuers negatively affects vital signs and physiological variables such as HR, blood lactate and cortisol. In other words, the use of a weighted vest increases metabolic and cardiovascular demands. In addition, the aging of health care workers, which could be associated with weight gain, may mirror these findings. Further studies are required to determine if the implementation of physical training programs could improve the CPR performance of healthcare professionals.

## Figures and Tables

**Table 1:** Descriptive data of the sample before CPR.

	Without Vest	With Vest	Odds Ratio	*p*-Value
	(*n* = 15)	(*n* = 16)	[95% Confidence Interval]	
Shift:				
Tomorrow	6 (40.0%)	9 (56.2%)	Ref.	Ref.
Late	9 (60.0%)	7 (43.8%)	0.53 [0.12; 2.26]	0.396
Age (years)	26.4 (9.3)	22.3 (3.8)	0.90 [0.77; 1.04]	0.151
Gender:				
Male	3 (20.0%)	7 (43.8%)	Ref.	Ref.
Female	12 (80.0%)	9 (56.2%)	0.34 [0.06; 1.65]	0.186
Weight (kg)	59.3 (9.8)	69.2 (17.0)	1.06 [0.99; 1.13]	0.073
Height (cm)	1.65 (0.1)	1.71 (0.1)	838 [0.20; 3,440,609]	0.113
Body mass index	21.7 (3.1)	23.5 (3.8)	1.17 [0.94; 1.47]	0.169
IPAQ:				
Sedentary	6 (40.0%)	12 (75.0%)	Ref.	Ref.
Active	4 (26.7%)	3 (18.8%)	0.40 [0.06; 2.48]	0.322
Sportsman	5 (33.3%)	1 (6.3%)	0.12 [0.00; 1.01]	0.051
Alcohol:				
No	11 (73.3%)	11 (68.8%)	Ref.	Ref.
Yes	4 (26.7%)	5 (31.2%)	1.23 [0.25; 6.51]	0.797
Tobacco:				
No	11 (73.3%)	13 (81.2%)	Ref.	Ref.
Yes	4 (26.7%)	3 (18.8%)	0.65 [0.10; 3.78]	0.631
Coffee:				
No	4 (26.7%)	5 (31.2%)	Ref.	Ref.
Yes	11 (73.3%)	11 (68.8%)	0.81 [0.15; 4.04]	0.797
Energy Drink:				
No	12 (80.0%)	12 (75.0%)	Ref.	Ref.
Yes	3 (20.0%)	4 (25.0%)	1.31 [0.23; 8.47]	0.764
Room temperature (°C)	21.2 (1.6)	21.3 (1.7)	1.05 [0.67; 1.65]	0.823
Room Humidity (%)	38.5 (5.8)	37.9 (6.3)	0.98 [0.87; 1.11]	0.753
Room Lumens (lm)	696 (18.8)	712 (39.5)	1.02 [0.99; 1.05]	0.176
Room Sound (dB)	72.0 (5.2)	72.3 (4.6)	1.01 [0.87; 1.18]	0.854
Oxygen saturation (%)	98.7 (1.2)	98.9 (1.2)	1.23 [0.65; 2.33]	0.517
Systolic blood Pressure (mmHg)	132 (21.5)	130 (14.6)	0.99 [0.95; 1.03]	0.749
Diastolic blood Pressure (mmHg)	76.7 (11.8)	76.2 (8.4)	1.00 [0.93; 1.07]	0.892
Mean blood Pressure (mmHg)	95.0 (14.2)	94.0 (9.8)	0.99 [0.93; 1.05]	0.813
Heart rate (beats/min)	75.2 (12.1)	85.1 (22.7)	1.03 [0.99; 1.08]	0.151
Temperature (°C)	36.2 (0.4)	36.3 (0.6)	1.84 [0.42; 7.99]	0.417
Pain scale (VAS)	0.13 (0.5)	0.19 (0.4)	1.32 [0.26; 6.63]	0.738
Lactate (mmol/L)	3.05 (1.7)	3.66 (1.3)	1.33 [0.80; 2.21]	0.268
Glucose (mg/dL)	96.9 (13.3)	102 (15.5)	1.03 [0.98; 1.09]	0.289
Cortisol (nmol/L)	184 (64.4)	261 (110)	1.01 [1.00; 1.02]	0.042
Right pupil size (mm)	3.37 (0.5)	3.50 (0.7)	1.42 [0.44; 4.65]	0.559
Left pupil size (mm)	3.35 (0.6)	3.41 (0.7)	1.18 [0.36; 3.84]	0.779

**Table 2:** Analysis of the effects of the vest and time on vital signs during CPR.

Variable	Vest Effect (*p*-Value)	Time Effect (*p*-Value)
Oxygen Saturation	*p* = 0.493	*p* = 0.008
Systolic Blood Pressure	*p* = 0.385	*p* < 0.001
Diastolic Blood Pressure	*p* = 0.727	*p* = 0.011
Mean Blood Pressure	*p* = 0.385	*p* < 0.001
Heart Rate	*p* = 0.001	*p* < 0.001
Body temperature	*p* = 0.111	*p* = 0.005

**Table 3:** Analysis of the effects of the vest and time on the physiological variables.

Variable	Vest Effect (*p*-Value)	Time Effect (*p*-Value)
Pain Scale (VAS)	*p* = 0.302	*p* = 0.015
Lactate	*p* < 0.001	*p* < 0.001
Cortisol	*p* = 0.014	*p* < 0.001
Glucose	*p* = 0.402	*p* = 0.242
Right pupillary size	*p* = 0.412	*p* < 0.001
Left pupillary size	*p* = 0.815	*p* < 0.001

## Data Availability

The datasets generated and/or analyzed during the current study are not publicly available owing to the inclusion of confidential data but are available from the corresponding author upon reasonable request.

## Abbreviations

The following abbreviations are used in this manuscript:

AHA	American Heart Association
ANOVA	Analysis of Variance
BMI	Body Mass Index
CCs	Chest Compressions
CPR	Cardiopulmonary Resuscitation
ERC	European Resuscitation Council
GERT	GERonTologic simulator
HR	Heart Rate
IPAQ	International Physical Activity Questionnaire
SESCAM	Castilla-La Mancha Health Service
SPO_2_	Oxygen saturation
VAS	Visual Analog Scale
